# Genome Sequences of Two *Enterobacter pulveris* Strains, 601/05^T^ (=LMG 24057^T^ =DSM 19144^T^) and 1160/04 (=LMG 24058 =DSM 19146), Isolated from Fruit Powder

**DOI:** 10.1128/genomeA.00991-13

**Published:** 2013-12-05

**Authors:** Gopal R. Gopinath, Christopher J. Grim, Ben D. Tall, Mark K. Mammel, Venugopal Sathyamoorthy, Larisa H. Trach, Hannah R. Chase, Séamus Fanning, Roger Stephan

**Affiliations:** CFSAN, FDA, Laurel, Maryland, USAa; UCD Centre for Food Safety, School of Public Health, Physiotherapy & Population Science, University College, Dublin, & WHO Collaborating Centre for Cronobacter, Belfield, Dublin, Irelandb; Institute for Food Safety and Hygiene, University of Zürich, Zürich, Switzerlandc

## Abstract

We report the draft genome sequences of the *Enterobacter pulveris* strains 601/05^T^ (=LMG24057^T^ =DSM19144^T^) and 1160/04 (=LMG24058 =DSM19146), isolated from fruit powder. The genome assemblies for the *E. pulveris* type strain, LMG24057, and strain LMG24058 have sizes of 4,708,624 and 4,811,103 bp and G+C contents of 56.6% and 56.5%, respectively.

## GENOME ANNOUNCEMENT

Stephan et al. (1) reported the isolation of six strains from fruit powder and from infant formula and its production environment, which were presumptively identified as *Enterobacter sakazakii*, now in the genus *Cronobacter*, through the use of differential chromogenic media. Additionally, these isolates displayed the yellow pigmentation on tryptone soy agar plates that is typical of *Cronobacter* (2). Using a typical polyphasic taxonomic scheme, Stephan et al. (1) classified these strains as belonging to the novel species *Enterobacter pulveris*.

Recently, Brady et al. (3) proposed that *E. pulveris* be recognized as a new *Cronobacter* species, and subsequently, Masood et al. (4) published a first draft genome sequence for *E. pulveris* strain E441 (=LMG24059). Because the taxonomic position remains unclear, we sequenced two strains of *E. pulveris*, the type strain 601/05 (=LMG24057 =DSM19144) and strain 1160/04 (=LMG24058 =DSM19146), which were originally described by Stephan et al. (1). The libraries were constructed using the Nextera XT DNA sample preparation kit (Illumina, San Diego, CA), and whole-genome sequencing was performed on a MiSeq sequencer (Illumina, San Diego, CA), utilizing 500-cycle paired-end version 2 chemistry. Paired-end FASTQ datasets were trimmed and assembled using the CLC Genomics Workbench, version 6.5 (CLC bio, Aarhus, Denmark). A draft genome of 4,708,624 bp, contained on 252 contigs (>500 bp in size), was obtained for strain 601/05^T^, while that of strain 1160/04 was 4,811,103 bp on 137 contigs (>500 bp in size). The genomic contigs were annotated using the RAST server (5) to identify RNAs and protein-coding genes. The draft genomes of strains 601/05^T^ and 1160/04 are predicted to contain 4,440 and 4,570 coding sequences (CDSs), respectively.

The two *E. pulveris* genomes share an average nucleotide identity of 98.98%. Both genomes possess a number of noteworthy features, namely, operons for the catabolism of protocatechuate, xylose, β-xyloside, sucrose, pentose sugar alcohol, l-rhamnose, d-galactarate, d-galactonate, d-serine, fructoselysine, sialic acid, 5-keto-d-gluconate, and l-idonic acid, as well as the presence of three type 1, one P, and one sigma chaperone-usher fimbria clusters, curli fimbriae, a *pga* biofilm operon, a CRISPR element, and the *lsr* autoinducer-2 operon. Additionally, each genome contains the uptake of hexose phosphates (*uhp*) system and a number of PTS- and ABC-type transporters of unidentified substrates.

There are also a number of genes and features that are unique to each genome, such as prophages and prophage-like elements and type VI secretion system cluster genes. Additionally, the genome of the type strain 601/05 harbors an α-xyloside and a β-linked disaccharide utilization operon, as well as two additional type 1 fimbria clusters. Conversely, the genome of strain 1160/04 contains a melibiose catabolism operon, a Tn*7*-like transposon harboring cobalt, cadmium, zinc, and mercury resistance, and an IncF class conjugative (*tra*) plasmid.

### Nucleotide sequence accession numbers.

The whole-genome shotgun projects for *E. pulveris* strains 601/05^T^ and 1160/04 are available in GenBank under accession no. AXSY00000000 and AXSZ00000000. The corresponding NCBI Biosample records, SAMN02369274 (tax ID, 1406823) and SAMN02369275 (tax ID, 1406822), are subject to taxonomic revision.
